# The Impact of the Brain Wave Modulation Technique (BMW-T) on Heart Rate Variability: A Single Session for Short-Term Stress Reduction

**DOI:** 10.3390/jcm14030715

**Published:** 2025-01-22

**Authors:** Marco Borgese, Luigi Tinella, Mauro Cozzolino, Giovanna Celia

**Affiliations:** 1 Department of Humanities, Philosophy and Education, University of Salerno, 84084 Fisciano, Italy; ltinella@unisa.it; 2 Department of Human Sciences, Education and Sport, Univesity Telematica Pegaso, 80100 Naples, Italy; giovanna.celia@unipegaso.it

**Keywords:** stress management, mindbody, clinical psychology

## Abstract

**Background**: Recent advances in psychophysiology have underscored the importance of autonomic nervous system modulation in managing short-term stress. While several interventions have demonstrated efficacy in reducing short-term stress and anxiety symptoms, there remains a gap in understanding how different short-term techniques compare in terms of both psychological and physiological outcomes. This study investigated the effects of a single session of the Brain Wave Modulation Technique (BWM-T) compared with a psychoeducational session on short-term stress management. **Methods**: A total of 72 university students participated in this study (mean age = 27.5 years, 79% female). They were randomly assigned to either an experimental group (n = 36) receiving BWM-T or a control group (n = 36) receiving a standard psychoeducational short-term stress management session. Pre- and post-intervention measures included HRV parameters, perceived stress (using the Distress Thermometer, DT), and anxiety (using the STAI-Y State Anxiety Scale, S-ANX). **Results**: Both groups experienced significant reductions in perceived stress (DT: MD = 1.42, *p* < 0.001) and anxiety (S-ANX: MD = 6.93, *p* < 0.001). However, only the experimental group demonstrated physiological changes indicative of improved autonomic function: decreased low-frequency (LF) power (MD = −0.369, *p* < 0.05) and a lower LF/HF ratio (MD = −1.09, *p* < 0.05). These findings highlight the unique physiological benefits of BWM-T, beyond the general psychological improvements seen in both interventions. **Conclusions**: BWM-T appears to be a promising, effective short-term intervention for reducing short-term stress and enhancing autonomic regulation. Further studies are needed to evaluate its long-term effects and potential for broader implementation.

## 1. Introduction

The COVID-19 pandemic has significantly impacted global stress levels, a phenomenon extensively documented by a growing body of research. According to the American Psychological Association (APA) [1], perceived stress is described as a physiological or psychological reaction triggered by internal or external stressors. This response leads to changes that can impact multiple body systems, shaping emotions, cognitive processes, and behaviors. The definition emphasizes the multifaceted nature of stress, integrating both physiological and psychological dimensions, which underscores its importance as a key topic in scientific research.

Studies [2,3,4,5,6] have highlighted the profound effects on psychological well-being, demonstrating an increase in stress levels compared with pre-pandemic times. The ongoing nature of the pandemic, coupled with economic uncertainties and geopolitical tensions, has exacerbated concerns about personal safety, financial stability, and overall well-being. These factors have notably impaired sleep quality and mental health, as shown by recent findings [7].

Short-term and long-term stress are known to negatively affect health [8]. While short-term stress responses can be adaptive, long-term stress may lead to significant health deterioration, including weakened immune function, increased risk of heart disease, and worsening symptoms of chronic conditions [9]. The impact of stress is influenced by the nature of the stressor, its duration, the individual’s biological vulnerability, and the available psychosocial resources [10].

A critical physiological parameter increasingly studied for its sensitivity to short-term stress is heart rate variability (HRV). HRV measures the temporal fluctuations between heartbeats and serves as an indicator of the autonomic nervous system’s ability to regulate cardiac function [11,12]. High HRV is associated with better health, whereas reductions in HRV are linked with prolonged stress exposure [13,14]. This relationship underscores the activation of the sympathetic nervous system under stress, which increases heart rate and decreases HRV [15].

Reduced HRV has been associated with a variety of physical and psychological disorders [13]. During extended periods of stress, the body remains in a state of heightened sympathetic activation, leading to increased heart rate and reduced HRV [14,15]. Thus, HRV is generally regarded as a sensitive indicator of autonomic nervous system regulation and the body’s capacity to respond to short-term and long-term stress. A reduction in HRV correlates with an elevated risk of cardiovascular disease, psychological disorders, and mortality.

Yoo [16] evaluated 40 university students during a regular academic period (low stress) and again during an examination period (high stress), focusing on key HRV parameters such as SDNN, RMSSD, and the LF/HF ratio. Their findings showed significant reductions in HRV (approximately 10–25% decreases in parameters like SDNN and RMSSD) when comparing the exam period with the low-stress period.

The restoration of heart rate to a resting state involves the activation of the parasympathetic branch of the autonomic nervous system. This activation, mediated by the vagus nerve, releases acetylcholine, a neurotransmitter that inhibits the sinoatrial (SA) node, thus decreasing heart rate [17]. Resting and self-regulated states are characterized by increased HRV, largely due to respiratory-driven parasympathetic control of heart rate, as slower breathing enhances vagus nerve activity [17]. HRV is thus used as an indicator of autonomic cardiac activity to assess continuous changes in parasympathetic function both at rest and under specific conditions [18].

Given the sensitivity of HRV to short-term stress, it is essential to employ methods capable of promoting short-term stress prevention and management by modulating HRV. Interventions that combine mental and physical well-being include a wide range of techniques, such as relaxation methods, hypnosis, visualization exercises, meditation, biofeedback, cognitive-behavioral therapies, group support sessions, and spiritual practices. Additionally, practices like yoga, tai chi, and qigong, which focus on movement, breath, and sound, are also effective in promoting self-awareness and reducing short-term stress and anxiety symptoms.

Endler [19] revisited the concepts of state and trait anxiety, emphasizing that state anxiety is a temporary emotional condition elicited by specific situations, whereas trait anxiety refers to a more enduring predisposition to respond with anxiety across various circumstances.

Despite their benefits, mind–body interventions have some limitations, such as the requirement for extensive training and time to master, as well as the need for regular practice and often group-based delivery [20,21]. In this study, we selected a short-term stress reduction method known as the brain wave modulation technique (BWM-T) [22,23,24,25,26,27], which has its roots in the interplay between Oriental doctrines and the scientific study of the mind–body dialogue [17,28,29].

The rationale behind the technique dates back to the experimental studies conducted the effects of Zazen practice on sleep and brainwaves [17,30,31,32].

BWM-T presents several advantages when compared with traditional mind–body techniques. To begin with, it is remarkably simple to master and can be completed in a short span of time. As a result, it becomes a sustainable and repeatable practice that students may find more appealing than alternative methods requiring greater time commitments and more complex learning processes. Secondly, no special premises or equipment are required, so BWM-T can be performed virtually anywhere. Thirdly, the intervention can be administered individually or to a large number of subjects simultaneously in a single session, as in this study, and requires only one psychologist, which simplifies planning. Moreover, once the technique is learned, subjects can perform it independently.

A previous study demonstrated the positive effect that BWM-T application can have in reducing the level of short-term perceived stress compared with other types of stress management practices, even with a single session [17,24,33,34,35].

The purpose of this study was to evaluate the impact of a single session of BWM-T on perceived short-term stress levels and anxiety symptoms levels, investigating the effects of BWM-T on heart rate variability (HRV). The impact of the BWM-T was compared with that of a control group that was subjected to a classic psychoeducation session.

In this study, our first hypothesis was that a single session of BWM-T will be accompanied by positive changes in heart rate variability (HRV) measures, indicating better regulation of the autonomic nervous system (ANS) in the experimental group compared with the control group undergoing a psychoeducation session. Also, as a second hypothesis, we expected significantly reduced perceived short-term stress and anxiety symptoms and levels in the experimental group compared with the control group.

## 2. Materials and Methods

### 2.1. Sample

Using G∗Power 3.1 [36], a power analysis was performed to determine the required sample size. The parameters included an alpha level of 0.05, a conservative effect size of 0.12, and a power of 0.80. The findings revealed that a sample of 56 participants would provide an 80% likelihood of correctly rejecting the null hypothesis. The sample was composed of non-clinical participants, including both undergraduate and postgraduate university students from various levels of education. Recruitment took place via an email announcement sent to all students through academic platforms, resulting in 72 individuals expressing interest in this study. Exclusion criteria included being under 18 years of age and having severe mental or physical disabilities, which led to all 72 respondents being deemed eligible. The average age of the participants was 27.5 years (SD = 13.48). Of those taking part, 57 were female, reflecting the typical demographic distribution of the university programs involved.

Each participant completed a questionnaire at two distinct time points, before and after the intervention, to gather demographic and psychological information. A unique subject ID was assigned to each individual. The informed consent form clearly stated that participants could withdraw from this study at any moment. All questionnaires were anonymous to ensure the data’s confidentiality and reliability. All procedures followed in this study complied with the ethical guidelines established by the research committee of the Italian Association of Psychology (AIP) and by the 1964 Helsinki Declaration and its subsequent amendments. The Ethics Commission of the University of Salerno (Unisa) approved this study on 17 February 2024 (protocol number 0064918).

### 2.2. Measures

All measures were digitized and made available to the participants via a shared computer link through an academic platform.

Perceived short-term stress was measured using the Distress Thermometer (DT) [37], a single-item screening tool known for its high sensitivity and specificity in capturing short-term stress [38]. This instrument has previously been validated for use in the Italian context [33,35]. Participants rated their current stress level on an 11-point scale from 0 (no distress) to 10 (maximum distress) [37]. Owing to its concise, single-question format, the DT is particularly suitable for inclusion in stress management research with university students.

Anxiety levels were measured using the State–Trait Anxiety Inventory Form Y (STAI-Y) [39], a self-report instrument that evaluates both present (state) anxiety and a generalized tendency toward anxiety (trait). The scale has demonstrated robust psychometric properties in Italian samples [40]. In this study, only the state subscale (S-ANX) was utilized, comprising 20 items. Each item is scored on a 4-point Likert scale (1 = “not at all” to 4 = “very much so”), yielding a total range of 20 (low anxiety) to 80 (high anxiety). Elevated scores correspond to greater anxiety levels tied to the participant’s immediate emotional state.

Heart rate data were collected via a blood volume pulse (BVP) sensor (Thought Technology, Montreal, Canada) attached to each participant’s index finger. Recordings were taken for five minutes to meet the standard minimum short-term requirements [17], followed by an additional five minutes post-intervention. For both the baseline and final measurements, a ProComp Infiniti device was used in conjunction with Biograph software (Thought Technology; Biograph software version 6.0.4). Inter-beat intervals underwent a power spectral analysis, using a 2000 ms high cutoff and a 300 ms low cutoff. The densities (ms^2^/Hz) of the high-frequency (HF; 0.15–0.4 Hz), low-frequency (LF; 0.04–0.15 Hz), and very-low-frequency (VLF; 0.016–0.04 Hz) bands were expressed as percentages over the full frequency range (0.016–0.500 Hz). Subsequently, RR (inter-beat interval) data were processed using Kubios HRV Premium (version 4.1.0; University of Eastern Finland, Kuopio, Finland). Preprocessing followed the software’s default pipeline, including artifact correction and resampling. The resulting data were converted into frequency domain parameters, low frequency (LF; both sympathetic and parasympathetic components), high frequency (HF; parasympathetic component), total power (TP; overall autonomic activity), and the LF/HF ratio (sympathetic activity), using a fast Fourier transform (FFT). To address variance discrepancies, TP, LF, and HF values (ms^2^/Hz) underwent natural log transformation.

### 2.3. Design

We used a randomized controlled trial design with one between-subject factor and one within-subject factor. Prior to the commencement of this study, each participant was provided with an informed consent form outlining the objectives, procedures, potential benefits and risks, and their right to withdraw from the research at any time without any consequences. All participants signed the form, confirming their willingness to take part in this study. Using a randomizer tool [41], a blocked randomization list was generated and subsequently applied to the sample [42]. Following this procedure, the participants were assigned either to the control or the experimental condition. In total, 36 individuals were allocated to the control condition and 36 to the experimental condition.

### 2.4. Intervention

The intervention was carried out in a single session for all participants. A script with research instructions was read to all participants in both the control and experimental groups. After completing the psychological questionnaires (STAI-Y S-ANX and DT), a BVP sensor was applied to all subjects to monitor HRV.

After application of the BVP sensor, all participants underwent a 5 min baseline to pre-estimate their HRV parameters. All participants were asked to close their eyes and breathe normally.

After this, the participants in the experimental condition received a 16 min session of the BWM-T, while the controls watched 16-min videos providing tips on short-term stress reduction.

In the experimental condition, given the relevance of mind–body interventions for short-term stress reduction emerging in the literature [18], we employed a specific intervention, the Brain Wave Modulation Technique (BWM-T), which has already demonstrated its effectiveness in several studies [22,23,24,25,26,27]. The technique involves a four-phase finger movement procedure that is easy to implement and spontaneously helps our brain release slower alpha waves [43].

The BWM-T protocol used in the experimental condition involved a 4-step finger movement.

The researcher, a clinical psychologist, described the technique and showed each of the four BWM-T finger positions, ensuring that the participants could easily mirror and learn them. The first position involved touching the tip of the little finger to the tip of the thumb; the second position, touching the tip of the ring finger to the tip of the thumb; the third position, touching the tip of the middle finger to the tip of the thumb; and the fourth position, touching the tips of the middle and ring fingers to the tip of the thumb. Participants were instructed to hold each position for at least 4 min, with the psychologist providing reminders to change positions. Afterwards, they were asked to place both hands on their legs or the armrest of the chair and close their eyes [33,35].

In the control group, participants watched a 15 min informational video on short-term stress management. In the video, a psychologist explained how stress develops and how it can negatively affect both body and mind, before presenting a set of practical coping strategies.

At the end of the sessions, all subjects were given a further 5 min measurement, during which they were asked to close their eyes and breathe normally. After this measurement, the BVP sensor was removed and the STAI-Y (S-ANX) and DT tests were administered again.

### 2.5. Statistical Analysis

Before conducting the analysis, the data were checked for missing values and outliers using box plots. No missing data were identified. Normality, linearity, and homoscedasticity were also assessed. Linearity was evaluated by calculating the Variance Inflation Factors (VIFs). Homoscedasticity (the assumption of equal variances across groups) was examined using bivariate scatter plots. While some variables were not normally distributed, none of the skewness and kurtosis values exceeded the critical thresholds outlined. Therefore, all variables were retained for analysis. Descriptive statistics were conducted to examine the characteristics of the sample.

A series of repeated measures 2 × 2 ANOVAs was performed, with the assessment phase (pre vs. post) and group (experimental vs. control) as independent variables, and the physiological measures (i.e., SDNN, rMSSD, VLF, LF, HF, LF/HF, pNN50, and HR) and psychological measures (i.e., STAI-Y S-ANX and DT) as dependent variables. Tukey’s post hoc comparisons were conducted for significant effects. Partial eta-squared was reported as a measure of effect size, and a *p*-value of 0.05 was set for statistical significance.

## 3. Results

### 3.1. SDNN

**Figure 1:**
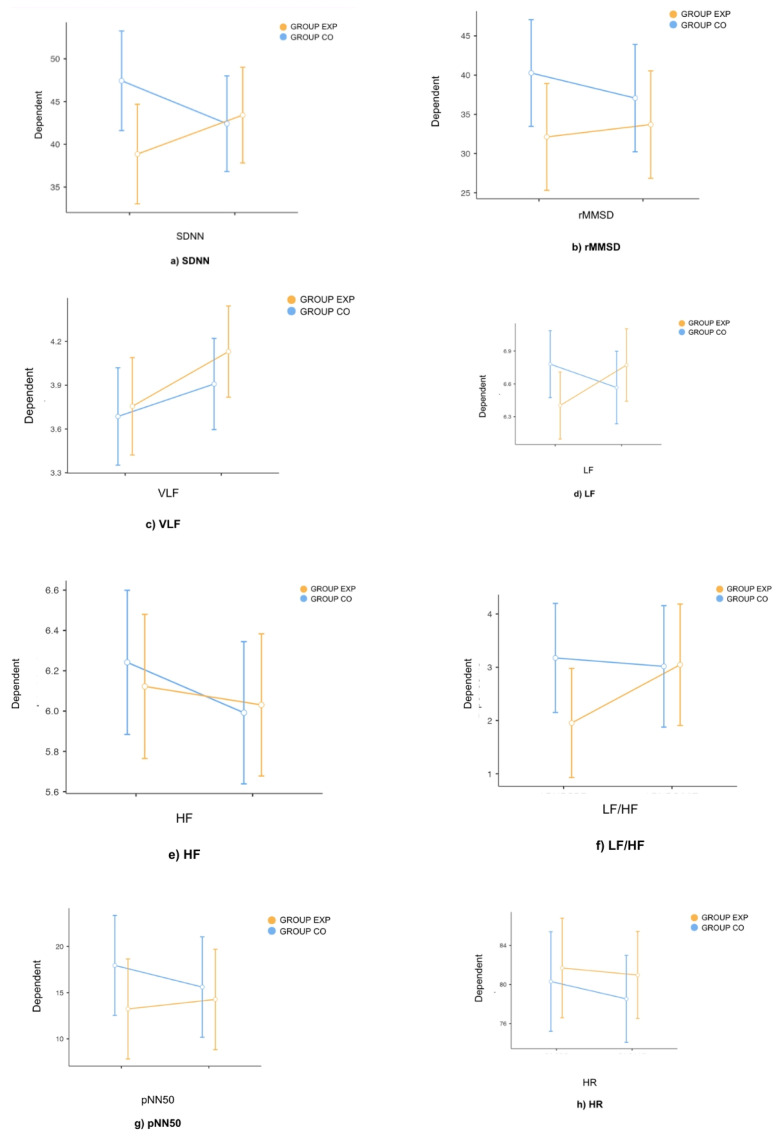
Comparison of heart rate variability (HRV) parameters in the experimental and control groups, before and after the intervention. Each panel represents a specific HRV parameter: (**a**) SDNN (standard deviation of NN intervals), (**b**) rMSSD (root mean square of the successive differences), (**c**) VLF (very-low-frequency power), (**d**) LF (low-frequency power), (**e**) HF (high-frequency power), (**f**) LF/HF ratio, (**g**) PNN50, and (**h**) HR (heart rate). The results indicate that the experimental group showed a significant reduction in the LF/HF ratio and an increase in LF (*p* < 0.05) compared with the pre-test. No significant differences were observed in the SDNN, RMSSD, VLF, HF, PNN50, or HR parameters between the pre- and post-test in either group.

The first repeated measures ANOVA was conducted considering the effects of the assessment phase and group belonging on the measure of SDNN. Significant results emerged for the effect of the interaction between the two independent variables, with a large effect size [F(1, 70) = 14.29; *p* < 0.001; η*p*^2^ = 0.170]. Tukey’s post hoc comparisons revealed that the participants in the control group showed a significantly higher SDNN in the pre-test than in the post-test (MD = 5.02; *p* < 0.001). No significant result emerged for the main effect of the assessment phase (see Table 1; Figure 1a).

The results can be described as follows. As regards the group factor, significant results emerged due to the interaction between the intervention and group to which participants belonged. Post hoc comparisons highlighted that in the control group, the SDNN measure was significantly higher in the pre compared with the post for the control group. In contrast, the SDNN measure increased non-significantly in the post-test compared with the pre-test in the experimental group.

### 3.2. rMSSD

The next repeated measures ANOVA was conducted considering the effects of the assessment phase and group belonging on the measure of rMSSD. Significant results emerged for the effect of the interaction between the two independent variables with a medium effect size [F(1, 70) = 8.44; *p* < 0.01; η*p*^2^ = 0.10]. Tukey’s post hoc comparisons revealed that participants in the control group showed significantly higher rMSSD in the pre-test than in the post-test (MD = 3.20; *p* < 0.05). No significant result emerged for the main effect of the assessment phase (see Table 1; Figure 1b).

The post hoc comparisons highlighted that the rMSSD measure was significantly higher in the pre compared with the post for the control group, while no difference between the assessment phases emerged for the experimental group.

### 3.3. VLF

A further repeated measures ANOVA was conducted considering the effects of the assessment phase and group belonging on the measure of VLF. Significant results emerged for the main effect of assessment phase, showing a medium effect size [F(1, 70) = 8.20; *p* < 0.01; η*p*^2^ = 0.10]. Tukey’s post hoc comparisons revealed that participants (in both groups) showed significantly higher VLF at the post-test than at the pre-test (MD = 0.299; *p* < 0.01). No significant result emerged for the interaction effect between independent variables (see Table 1; Figure 1c).

Thus, significant results emerged for the effect of the assessment phase on VLF. The analysis of the post hoc comparisons revealed that in both groups, the VLF measure was significantly higher in the post-test compared with the pre-test.

### 3.4. LF

A further repeated measures ANOVA was conducted considering the effects of the assessment phase and group belonging on the measure of LF. Significant results emerged for the interaction effect between independent variables, showing a medium effect size [F(1, 70) = 9.76; *p* < 0.01; η*p*^2^ = 0.12]. Tukey’s post hoc comparisons revealed that participants in the experimental group showed significantly higher LF at the post-test than at the pre-test (MD = −0.369; *p* < 0.05). No significant result emerged for the main effect of the assessment phase (see Table 1; Figure 1d).

Significant results emerged due to the interaction between the intervention and the group on the LF parameter. Post hoc comparisons showed a significant difference between the post and pre measures in the experimental group: the participants of the experimental group had a significantly lower mean LF measure in the pre compared with the post. The participants of the control group showed a reduction in the measure in the post compared with the pre, which, however, is not significant.

### 3.5. HF

Another repeated measures ANOVA was conducted considering the effects of the assessment phase and group belonging on the measure of HF. Significant results emerged for the main effect of assessment phase, showing a medium effect size [F(1, 70) = 5.31; *p* < 0.05; η*p*^2^ = 0.07]. Tukey’s post hoc comparisons revealed that participants (in both groups) showed a significantly higher HF at the pre-test than at the post-test (MD = 0.171; *p* < 0.05). No significant result emerged for the interaction effect between independent variables (see Table 1; Figure 1e).

Significant results emerged for the effect of the intervention factor on the HF. The analysis of the post hoc comparisons reveal that in both groups, the HF measure is significantly higher in the pre compared with the post. The difference between the pre and post HF is significant and is significantly reduced in the post compared with the pre in both groups without any difference.

### 3.6. LF/HF

The next repeated measures ANOVA was conducted considering the effects of the assessment phase and group belonging on the measure of LF/HF. Significant results emerged for the effect of the interaction between the two independent variables, with a medium effect size [F(1, 70) = 5.13; *p* < 0.05; η*p*^2^ = 0.07]. Tukey’s post hoc comparisons revealed that the participants in the experimental group showed a significantly higher LF/HF in the post-test than in the pre-test (MD = −1.09; *p* < 0.05). No significant result emerged for the main effect of the assessment phase (see Table 1; Figure 1f).

Significant results emerged for LF/HF due to the interaction between the intervention and group belonging. Post hoc comparisons showed a significant difference between the post and pre measures in the experimental group; the participants of the experimental group had a significantly lower mean LF/HF measure in the pre compared with the post.

### 3.7. pNN50

The subsequent repeated measures ANOVA was conducted considering the effects of the assessment phase and group belonging on the measure of pNN50. Significant results emerged for the interaction effect between the independent variables, showing a medium effect size [F(1, 70) = 7.87; *p* < 0.01; η*p*^2^ = 0.10]. Tukey’s post hoc comparisons revealed that participants in the control group showed a significantly lower pNN50 at the post-test than at the pre-test (MD = 2.34; *p* < 0.05). No significant result emerged for the main effect of assessment phase (see Table 1; Figure 1g).

From the post hoc comparisons a significant difference emerged between the post and pre measures in the control group: the participants of the control group had a higher mean pNN50 measure than in the post, while no difference between the assessment phases emerged for the experimental group.

### 3.8. HR

The subsequent repeated measures ANOVA was conducted considering the effects of the assessment phase and group belonging on the measure of heart rate. Significant results emerged for the main effect of the assessment phase, with a small effect size [F(1, 70) = 4.16; *p* < 0.05; η*p*^2^ = 0.06]. Tukey’s post hoc comparisons revealed that participants (in both groups) showed significantly lower heart rates at the post-test than at the pre-test (MD = 1.25; *p* < 0.05). No significant result emerged for the interaction effect between the independent variables (see Table 1; Figure 1h).

### 3.9. STAI-Y S-ANX

**Figure 2:**
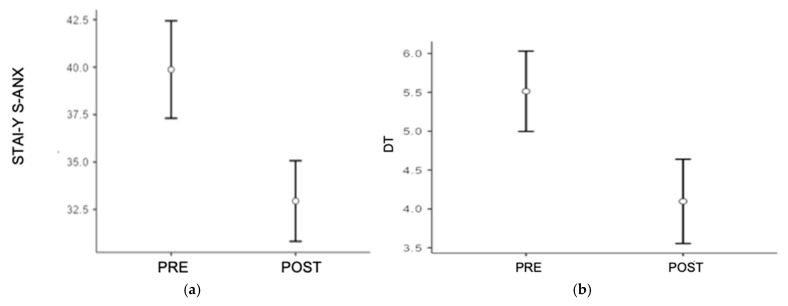
(**a**) Mean scores on the State–Trait Anxiety Inventory (STAI-Y S-ANX) for the experimental and control groups before and after the intervention. The results show a significant reduction in anxiety symptoms and levels for both groups post-intervention (*p* < 0.001), with no significant interaction effects between the groups. (**b**) Mean scores on the Distress Thermometer (DT) for the experimental and control groups before and after the intervention. Both groups exhibited significant reductions in perceived short-term stress post-intervention (*p* < 0.001), with no significant interaction effects between the groups.

A repeated measures ANOVA was conducted considering the effects of the assessment phase and group belonging on the measure of state STAI-Y (S-ANX). Considering the measure of state anxiety, significant results emerged for the main effect of assessment phase, showing a large effect size [F(1, 70) = 45.99; *p* < 0.001; η*p*^2^ = 0.39]. Tukey’s post hoc comparisons revealed that participants (in both groups) showed significantly higher state anxiety at the pre-test than at the post-test (MD = 6.93; *p* < 0.001). No significant result emerged for the interaction effect between the independent variables (see Table 2; Figure 2a).

### 3.10. DT

Finally, a repeated measures ANOVA was conducted considering the effects of the assessment phase and group belonging on the measure of DT. Significant results emerged for the main effect of assessment phase, showing a large effect size [F(1, 70) = 45.26; *p* < 0.001; η*p*^2^ = 0.39]. Tukey’s post hoc comparisons revealed that participants (in both groups) showed significantly higher scores for DT at the pre-test than at the post-test (MD = 1.42; *p* < 0.001). No significant result emerged for the interaction effect between the independent variables (see Table 2; Figure 2b).

The findings of this study indicate a significant reduction in STAI-Y (S-ANX) and DT scores in both the experimental and control groups when comparing the post-intervention with the pre-intervention measurements. This suggests that both types of intervention, BWM-T and the psychoeducational session, were effective in reducing perceived anxiety symptoms and stress in the short term.

## 4. Discussion

The present study aimed to investigate the impact of a single session of a mind–body intervention (BWM-T), compared with a psychoeducation session, on heart rate variability (HRV), perceived short-term stress, and anxiety symptoms.

It is worth highlighting that the sample was mostly composed of young adults who may have a parasympathetic reserve that limits the changes in autonomic nervous system activity, as has emerged in some studies [44,45].

In relation to the first hypothesis, the results show that in the experimental group, a single session of the mind–body intervention (BMW-T) produced statistically significant variations in two HRV indices, namely the LF/HF ratio and the LF, compared with the control group, where a psychoeducation session was used. The experimental group showed a significant reduction in the LF/HF ratio and LF indices. These reductions indicate a possible improvement in autonomic modulation, with a greater balance toward parasympathetic activity. This phenomenon has been well documented in the literature as indicative of short-term stress reduction [17]. Similarly, the significant reduction in the LF values suggests a decrease in sympathetic influence, consistent with a more controlled short-term stress response [45,46].

While we found differences between experimental and control group in the pre and post results for the LF/HF ratio and LF parameters after BWM-T, no differences were found for factors like SDNN, rMSSD, VLF, and HF. It is possible to hypothesize that a single session of BWM-T may not be sufficient to influence these HRV parameters because it does not act directly through breathing inducing the effect of respiratory sinus arrhythmia, which is capable of producing significant short-term effects on HRV through known mechanisms of action [11,17,47].

Regarding our second hypothesis, the results show a significant reduction in levels of perceived stress (DT) and anxiety symptoms (STAI-Y, S-ANX) in both groups (experimental and control). This result can be attributed to several factors.

Firstly, simply participating in a short-term stress management program could have induced a positive effect due to greater awareness of one’s psychological state and personal well-being [48]. The hypothesis that simply participating in a short-term stress management program, regardless of the specific technique used, can have positive effects is supported by several studies [49].

Secondly, other studies [19,48] highlight that although psychoeducational interventions can initially reduce perceived short-term stress as much as mind–body interventions, the latter produce long-term maintenance of the acquired benefits.

Finally, the number of subjects involved in this study could also have generated differences in terms of the outcome between psychological and physiological measures. In fact, while physiological measures require a lower number of subjects to have reliable results, self-reported psychological measures may require a higher number of participants [50]. Other studies show, in fact, that the use of the BWM-T on a greater number of participants [24,34] allows for more reliable results also in relation to psychological measures.

### Limitation and Future Directions

This study has several limitations that should be considered when interpreting its findings. Although this study presents adequate statistical power, the number of subjects should be expanded by including a clinical sample or people from different age groups and cultural backgrounds. Additionally, the effects of the BWM-T intervention were only measured immediately post-intervention. The lack of a long-term follow-up means that we have limited understanding of the intervention’s lasting impact on heart rate variability (HRV), stress levels, and potential benefits. This limitation highlights the importance of conducting longitudinal research in future studies to evaluate the sustainability of BWM-T’s effects over time. Such studies could include follow-up assessments over weeks or months and explore the potential cumulative effects of repeated intervention sessions.

Given that some psychological tests may not be as sensitive at low numbers, one limitation is that this study did not involve a larger number of subjects. Another limitation is the use of self-reported measures for perceived short-term stress and anxiety symptoms, such as the Distress Thermometer and STAI-Y (S-ANX).

Furthermore, this study did not account for some variables that could influence HRV, such as physical activity or caffeine intake before sessions. There are also technical limitations to consider. Although HRV is a reliable measurement, specific settings, equipment used, and cutoff frequencies in HRV analysis can affect the results and their interpretation.

Subsequent studies should overcome the limitations described by conducting longitudinal research to evaluate the long-term effects of BWM-T, even employing multiple sessions over an extended period of time. This would help to better understand the real impact of long-term stress reduction and improvements in autonomic regulation.

While the present study focused on a non-clinical population to explore the method’s effectiveness in a healthy demographic, clinical populations often present unique physiological and psychological characteristics, such as higher baseline short-term stress levels, compromised autonomic function, or chronic anxiety symptoms. Evaluating the effects of BWM-T within such populations could help determine its efficacy as a complementary therapeutic tool in clinical settings, such as for patients with anxiety disorders, depression, or stress-related somatic conditions. This would not only broaden the applicability of the intervention but also contribute to its validation as a targeted clinical strategy.

Further studies could consider comparing the BWM-T with other mind–body interventions to evaluate not only their benefits but also any advantages in terms of usability.

Finally, the inclusion of other physiological markers, such as cortisol levels, systolic blood pressure (SBP), or other stress biomarkers, could complement the HRV data and provide a more complete assessment of the physiological impact of the intervention.

## 5. Conclusions

In conclusion, this study confirms that a single session of the Brain Wave Modulation Technique (BWM-T) can produce significant changes in some heart rate variability (HRV) parameters, such as the LF/HF ratio and LF. These findings align with studies supporting the benefits of mind–body interventions for short-term stress reduction and autonomic regulation.

The hypothesis of a significant reduction in the levels of perceived short-term stress and perceived anxiety symptoms in the experimental group compared with the control group was not confirmed. In fact, both groups showed a statistically significant reduction between the pre and post measurements.

The lack of significant differences between the groups (BWM-T and psychoeducational) suggests that both approaches may be beneficial in the short term. However, BWM-T showed unique physiological benefits, which may be promising for long-term autonomic regulation and stress resilience.

This study’s limitations include a small sample and the lack of long-term follow-up, which restricts the ability to generalize the findings and understand the enduring effects of BWM-T. Future research should explore the impacts of repeated sessions over time and assess these effects within different populations to determine the sustainability and broader applicability of BWM-T as a short-term stress management tool. Additionally, the incorporation of other physiological stress markers could provide a more comprehensive view of the intervention’s effects. 

## Figures and Tables

**Table 1:** Main effects of time and interaction effects between time and groups for the ANOVA model performed on HRV parameters. The table presents the sum of squares (SS), degrees of freedom (DF), mean squares (MS), F-statistic (F), and *p*-values for the physiological measures SDNN, rMSSD, VLF, LF, HF, LF/HF, PNN50, and HR. Significant results (*p* < 0.05); *p* = significance level.

	SS	DF	MS	F	*p*
SDNN					
TIME	1.94	1	1.94	0.0335	0.855
TIME*GROUP	827.04	1	827.04	142.985	<0.001
Residuals	4048.88	70	57.84		
rMSSD					
TIME	23.7	1	23.7	0.976	0.327
TIME*GROUP	205	1	205	8.442	0.005
Residuals	1699.5	70	24.3		
VLF					
TIME	3.210	1	3.210	8.208	0.006
TIME*GROUP	0.210	1	0.210	0.537	0.466
Residuals	27.375	70	0.391		
LF					
TIME	0.218	1	0.218	0.695	0.407
TIME*GROUP	3.063	1	3.063	9.767	0.003
Residuals	21.950	70	0.314		
HF					
TIME	1.051	1	1.051	5.31	0.024
TIME*GROUP	0.226	1	0.226	1.14	0.289
Residuals	13.849	70	0.198		
LF/HF					
TIME	7.86	1	7.86	2.86	0.095
TIME*GROUP	14.09	1	14.09	5.13	0.027
Residuals	192.16	70	2.75		
pNN50					
TIME	15.5	1	15.5	1.20	0.278
TIME*GROUP	102.2	1	102.2	7.87	0.007
Residuals	909.0	70	13.0		
HR					
TIME	56.2	1	56.2	4.168	0.045
TIME*GROUP	10	1	10	0.743	0.392
Residuals	944.7	70	13.5		

**Table 2:** Main effect of time and interaction effect between time and groups for the ANOVA model performed on STAI Y S-ANX and DT. SS, sum of squares; DF, degrees of freedom; MS, mean of squares, residuals; F, F-statistic; *p* = significance level.

	SS	DF	MS	F	*p*
STAI Y–S-ANX					
TIME	1729.2	1	1729.2	45.99	<0.001
TIME*GROUP	45.6	1	45.6	1.21	0.275
Residuals	2631.8	70	37.6		
DT					
TIME	72.250	1	72.250	45.268	<0.001
TIME*GROUP	0.0278	1	0.0278	0.0174	0.895
Residuals	111.722	70	1.596		

## Data Availability

The raw data supporting the conclusions of this article will be made available by the authors on request.
